# Magnesium Supplementation and Blood Pressure in Pregnancy: A Double-Blind Randomized Multicenter Study

**DOI:** 10.1155/2018/4843159

**Published:** 2018-05-29

**Authors:** Maria Bullarbo, Helena Mattson, Anna-Karin Broman, Natalia Ödman, Thorkild F. Nielsen

**Affiliations:** ^1^Department of Obstetrics and Gynecology, Sahlgrenska Academy, University of Gothenburg, Gothenburg, Sweden; ^2^Department of Gynecology, Närhälsan, Mölndal, Sweden; ^3^Women's Clinic, Södra Älvsborgs Hospital, Borås, Sweden; ^4^Women's Clinic, Norra Älvsborgs Hospital, Trollhättan, Sweden

## Abstract

**Objective:**

To investigate the effect of magnesium (Mg) supplementation in healthy pregnant women for prevention of blood pressure increase. Secondary outcomes were comparison of biomarkers for hypertensive disorders and labour and fetal outcomes between the groups.

**Methods:**

Two hundred nulliparous healthy pregnant women were double-blind randomized to receive Mg daily or placebo.

**Results:**

There were no differences in blood pressure increase. However, among the Mg-treated women, there was a significant negative correlation between increase in blood levels of magnesium and increase in systolic blood pressure (*p* = 0.042). Magnesium supplementation seems to be safe for both mother and infant.

**Conclusion:**

Magnesium supplementation in healthy first-time pregnant women is not to be recommended for prevention of blood pressure increase. Supplementation in risk pregnancies needs to be further investigated. The study is listed on the ISRCTN registry with study ID 13890849.

## 1. Introduction

High blood pressure during pregnancy is a risk factor for developing preeclampsia (PE) and eclampsia (E) and affects approximately 6–8% of all pregnant women [1]. A systolic/diastolic blood pressure (SBP/DBP) of 140/90 mm Hg is defined as gestational hypertension (HT). The etiology of HT is multifactorial, but nulliparity, obesity, stress, heredity, high maternal age, multiple pregnancy, diabetes, thrombophilia, kidney disease, chronic HT, and nutritional deficiency are all risk factors [2]. When HT is accompanied by proteinuria of at least 0.3g/day, it is defined as PE. PE can be complicated by elevated liver enzymes, low platelets, or changes in the coagulation system, which are life-threatening conditions. PE can further lead to general convulsions, called eclampsia (E), for which the only known curative treatment is termination of the pregnancy. The mechanisms underlying the development of HT and PE are still largely unknown and seem to be multifactorial, involving both placental pathology and immunological or genetic disorders [3, 4]. If HT is complicated by PE, oxidative stress occurs, followed by the release of inflammatory mediators such as IL-6 [5–7]. Blood levels of both the renal marker cystatin C and the inflammatory marker CRP have also been shown to be elevated in women with hypertensive disorders [8–10]. Some studies have supported a link between HT and/or PE and deficiencies in calcium, vitamins C, D, and E [11–14], and folic acid [12, 15], but the evidence is not conclusive. However, a daily low dose of antiplatelet agents, such as aspirin, of at least 100 mg in risk pregnancies has in recent years been shown to prevent PE and intrauterine growth restriction (IUGR) [16–18]. It is important to note that most studies have focused on prevention of PE and not of gestational HT. There are very few, if any, human studies on the prevention of gestational HT, and, in fact, an animal study demonstrated contradictory results, showing that low-dosage aspirin treatment led to increased risk of HT [19]. It is risky to draw conclusions about effects in humans based on an animal study, but the results do indicate a need for further research on possible preventive agents against gestational HT.

Several studies suggest that the risk of gestational HT is related to changes in magnesium (Mg) homeostasis. Associations have also been reported between mortality in cardiovascular disease and Mg intake in nonpregnant populations [20]. Mg is one of the five most common minerals in the human body and is present in more than 300 human enzymes. Mg plays a major role in the normal functioning of muscles, carbohydrate metabolism, and the skeletal structure [21, 22]. In a Cochrane meta-analysis of Mg supplementation in pregnant women, Makrides et al concluded that it had not been proven to be efficient in preventing gestational HT, but many of the studies were classified as low quality [23] and more studies were therefore recommended. Of interest is the fact that one study showed a correlation between low plasma levels of Mg in pregnant women and PE [24]. The same study also showed that 16% of all pregnant women had low plasma levels of Mg. After the publication of the Cochrane review by Makrides, our research group demonstrated a positive correlation between the urinary secretion of Mg and calcium in early pregnancy and BP increase in late pregnancy [25]. In a follow-up double-blind randomized placebo-controlled study of 60 pregnant women, 300 mg Mg or placebo was administered daily as a supplement from gestational week 25 until labour. Women included were classified to belong to a risk group for developing BP increase due to high urinary secretion of calcium/Mg. The DBP increase during late pregnancy was significantly lower in the study group receiving Mg compared to the placebo group [26]. The same study showed that the expression of Mg-sensitive genes was also related to SBP and DBP and to Mg excretion in the urine. The results suggested that Mg is involved in the regulation of BP during pregnancy [27]. The same conclusion was reached by Rylander in a review of Mg and BP in pregnancy [28]. A retrospective study comparing women with and without PE also showed that an increase in DBP of ≥15 mm Hg was a risk indicator for developing PE, indicating that preventing gestational HT might also prevent the development of PE [29]. As early as 1998, it was shown that lowered red cell Mg concentrations were correlated to severe HT in pregnancy, suggesting that Mg deficiency could be a contributory factor in the development of hypertensive disorders of pregnancy [30]. The association of a low dietary Mg intake and an increased risk of PE has also been confirmed in a meta-analysis studying the effects of various dietary factors on the risk of pregnancy-induced high BP [31]. Spätling et al. concluded in a review on Mg and pregnancy that the need for Mg intake increases during pregnancy, with a daily recommended dose of supplemented Mg of 240–480 mg [32].

## 2. Objective

The primary aim of the study was to investigate whether a daily supplementation with 400 mg Mg during pregnancy compared to a placebo group in a double-blind setting could prevent an increase of diastolic BP of at least 15 mm Hg. Secondary outcomes were comparison of biomarkers for hypertensive disorders and labour and fetal outcomes between the groups.

## 3. Methods

The study was a placebo-controlled double-blind interventional multicenter study. A total of 199 nulliparous women in gestational weeks 12–14 were recruited at 3 antenatal care units (ACU) in west Sweden (in the cities of Borås, Alingsås, and Trollhättan). Inclusion criteria were nulliparity, no regular medication, normotension, singleton pregnancy, and maternity age >18 years and <40 years. Exclusion criteria were age <18 or >40 years, multiple pregnancies, trombophilia, previous labour, diabetes, chronic HT, kidney disease, heart disease, regular medication, history of cardiac arrhythmia, or heredity of sudden cardiac arrest. Abdominal ultrasound examination was performed for dating and verification of singleton and viable pregnancy. After oral and written consent, participants were randomized in a computerized double-blind procedure to receive either Mg (400 mg Magnesium Extra, Diasporal®) or placebo. The code was not broken until all participants had given birth. Blood samples were collected at gestational weeks 12–14 and 35 for analysis of IL-6, CRP, urate, cystatin C, Mg, Ca, albumin, creatinine, and glomerular filtration rate (GFR). BP was measured at the ACU at 2-3 weeks' intervals throughout the pregnancy, with the women seated with arm and backrest support, down to Korotkoff V with a manual sphygmomanometer. BP data registered at the ACU and labour ward were collected from medical records. Records were obtained on gestational length at birth, labour outcomes including excessive bleeding >1000 ml, instrumental delivery, and duration of active labour, and fetal outcomes including Apgar score at five minutes, pH in the arterial umbilical cord, birth weight, and need of care at a neonatal intensive care unit (NICU). Possible additional multivitamin intake containing extra Mg obtained from medical records was also registered.

## 4. Statistical Analyses

A sample size of 178 women was estimated to achieve 80% power at 5% significance level, assuming that 25% of Mg-treated women experience an increase of <15 mm Hg in DBP during pregnancy compared to 45% in the placebo group. This hypothesis was based on results from the first interventional pilot study of 60 women. Due to expected dropout in the study due to miscarriage or nausea, 200 women were calculated to participate in the study, of whom 100 women received Mg and 100 women received placebo. A *p* value < 0.05 was interpreted as statistically significant. For differences between the groups, the Mann–Whitney *U* test and Fisher's exact test were used. Spearman's test was used to analyze correlations between different variables. Mean (SD)/median (min; max) were used for descriptive purposes.

## 5. Results

For all outcomes regarding BP, labour, and infants' analyses, statistics on both intention to treat (ITT) and per protocol (PP) were calculated. As there were no statistically significant differences in any outcomes between ITT and PP groups, results for mainly the PP groups are described. The study had a dropout rate of 11% (7 women in the placebo group and 16 in the Mg group). Main reasons were nausea and difficulty in intake of the randomized supplementation. One exclusion occurred in the Mg group before randomization because of accidental damage of the randomized supplement, and therefore a total of 199 women were randomized (99 to the Mg group and 100 to the placebo). Baseline data were equal between the two study groups (Table 1). As there was no difference between the groups of women taking additional supplements of Mg, no regression analysis was performed.

**Figure 1:**
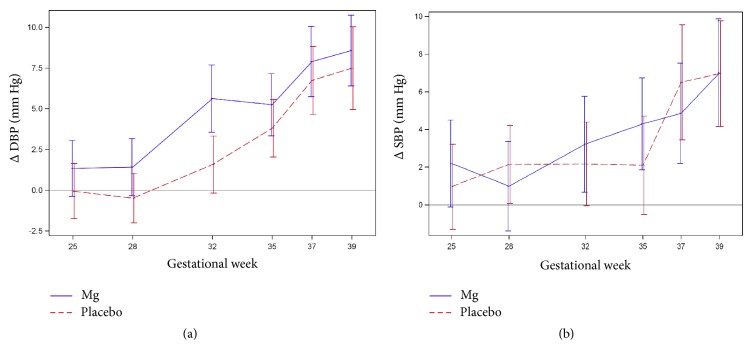
(a) Change (Δ) in DBP during pregnancy (PP). (b) Change (Δ) in SBP during pregnancy (PP).

**Figure 2:**
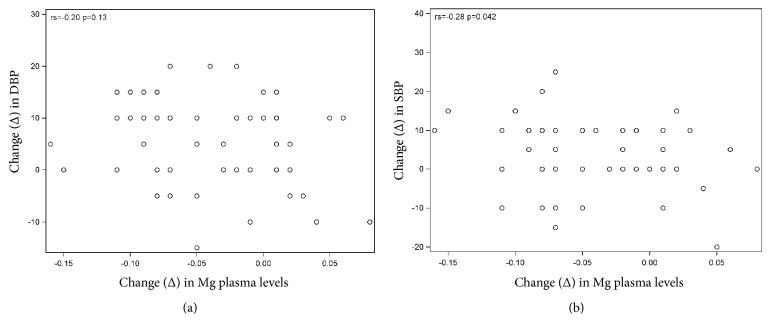
(a) Mg group. Spearman's correlation between change in DBP from week 12 to week 35 and change in Mg plasma levels from week 12 to week 35. (b) Mg group. Spearman's correlation between change in SBP from week 12 to week 35 and change in Mg plasma levels from week 12 to week 35.

The main outcome was BP increase, illustrated in Tables 2(a) and 2(b) (Table 2(b) only including women taking no extra Mg) and in Figures 1(a) and 1(b). For the primary outcome, there was no difference between the groups regarding increase in DBP or SBP. The number of women diagnosed with gestational HT and PE was equally distributed between the Mg and placebo groups (17 versus 20 and 3 versus 4, resp.). As regards secondary outcomes (Table 3), there were no differences in fetal or labour outcomes except for gestational length at birth (*p* = 0.03 for PP and 0.048 for ITT). Mean gestational length at birth was 40,2 (Mg) versus 39,9 weeks (placebo). Blood parameters were equal between the groups at gestational week 12, at week 35, and in change over time from week 12 to week 35 (Δ DBP and Δ SBP, Table 4). Scatter plots with Spearman's correlation showed no correlations between blood parameters or change in blood parameters and SBP or DBP (no table shown). However, when analyzing only the Mg group regarding correlations between Δ Mg levels from week 12 to week 35 and Δ SDP and DBP, there was a negative correlation regading SBP (*p* = 0,042) but not regarding DBP (*p* = 0,13), shown in Figures 2(a) and 2(b). It was not possible to analyze Mg-deficient women as they were too few (only 2 in the Mg group and 5 in the placebo group).

Of 199 women included in the study, 35% in the Mg group and 37% in the placebo group were supplemented with additional multivitamin tablets containing Mg. The doses of extra Mg varied between 30 and 150 mg/day.

## 6. Discussion

Healthy first-time pregnant women with no risk factors for developing gestational HT seem to have no need of extra daily supplementation of Mg to protect against BP increase. Therefore, a general recommendation of oral Mg supplementation during pregnancy remains controversial, despite an increased need for Mg during pregnancy. However, these results are somewhat unexpected, as our research group in earlier studies found correlations between supplementation with Mg and prevention of DBP increase. It must be emphasised, however, that the earlier promising results were achieved among pregnant nulliparous women with high urinary excretion of Mg and calcium in early pregnancy, indirectly indicating Mg deficiency. Participants in our earlier study were accordingly classified to belong to a risk group for developing BP increase during pregnancy.

Excretion of Mg is increased during stress reactions, including merely the state of being pregnant. The major question in this study is whether the participants were deficient in Mg or not. If not, they would be unlikely to benefit from supplementation with Mg, as the results indicate in this study. It is established that Mg deficiency is difficult to measure, as only 1% of all Mg is measurable in the blood, and blood levels decrease only when the deficiency is very serious. Hence, normomagnesemia does not exclude Mg deficiency [33]. Interpreting the results of biomarkers was difficult, as only two-thirds of the 199 participants gave a blood test in the third trimester. Despite this fact, it was unexpected to find no difference between the groups in plasma levels of Mg in the third trimester of pregnancy, despite differences in Mg intake.

The main shortcoming of the study was the fact that the participants were allowed to take multivitamin tablets containing Mg, which in general is recommended for pregnant women. Also, given current knowledge regarding increased need for Mg during pregnancy, performing a placebo-controlled study is hard to justify from an ethical point of view. However, they were not allowed to take extra Mg on their own as a supplement without reporting that to the study group, which no one did, nor was any registration of such use seen in the medical records. Furthermore, the possibility cannot be excluded that, after being informed about the study, an additional unknown number of participants took over-the-counter Mg. Consequently, there could be a treatment bias. Notably, however, Makrides, who authored the Cochrane meta-analysis on Mg supplementation during pregnancy, classified the Sibai study as high quality, despite the fact that it allowed extra Mg intake [34]. Another possible explanation to equal plasma levels of Mg between the study groups is that the bioavailability of Mg supplementation could be changed during pregnancy, and plasma Mg level is in fact a poor indicator of the total body magnesium content and availability. To our knowledge, studies on the bioavailability of multivitamin tablets during pregnancy have not been performed, and hence the possible impact on the results of such intake is unknown. Unfortunately, approximately 1/3 declined to leave blood samples in pregnancy week 35, most likely because the women had to pay an extra visit to the laboratory at a nearby hospital. To conclude, results from our study on Mg supplementation should be interpreted with caution.

Among secondary outcomes, there were statistical differences regarding gestational length and modes of delivery. These results should also be interpreted with caution given the small number of cases in our study, especially as our results are not confirmed by earlier studies summarized in the Cochrane review by Makrides [23]. There were no differences in fetal outcomes, indicating that Mg supplementation seems to be safe for the fetus. However, it would have been a strength to have also measured umbilical levels of Mg, as more studies are needed on possible effects in infants.

The strength of this study is that it is a randomized double-blind placebo-controlled trial that was completely blinded for the study group as well, since the randomization was performed by the manufacturing company and kept secret until onset of data collection. According to baseline data, the study groups were equivalent, with no statistical differences between groups (Table 1). The study protocol was followed correctly, the primary and secondary aims are clearly defined, and power calculation was performed. The number of dropouts was in line with power calculations (11%). The causes of dropout were registered and mainly linked to side effects. The study group was completely independent from the manufacturing company and had no conflicts of interest. Another important strength of the study is that Mg citrate was used for supplementation, with proven good bioavailability [35].

Finally, it must be underlined that research on the prevention of HT disorders during pregnancy is mainly focused on risk pregnancies. For instance, aspirin is only recommended to pregnant women at risk of developing PE or intrauterine growth restriction (IUGR). Results from our studies on Mg supplementation suggest that pregnant women with risk factors for developing hypertension disorders could benefit from extra Mg intake but are less likely to benefit if they have no risk factors. It is consequently reasonable to suggest that pregnant women at risk to a greater extent have Mg deficiency.

## 7. Conclusion

Mg supplementation during pregnancy seems to be safe for mother and infant and is inexpensive but is yet not proven to be effective in preventing BP increase among healthy nulliparous women. Interestingly, however, Mg-treated women who had an increase in plasma levels of Mg in fact had a prevention of SBP inrease. It is possible and even likely that women at risk of developing gestational HT or being Mg-deficient could benefit from Mg supplementation. Further studies are needed.

## Figures and Tables

**Table 1:** Baseline data (PP).

Variable	Magnesium (*n* = 83)	Placebo (*n* = 93)	*p* value
Maternal age	27.0 (3.4)	26.9 (3.5)	0.92
	27.0 (20.0; 36.0)	27.0 (18.0; 36.0)	
	*n* = 83	*n* = 93	

BMI (body mass index)	24.7 (4.7)	24.1 (3.8)	0.62
	24.0 (18.0; 43.0)	24.0 (18.0; 35.0)	
	*n* = 83	*n* = 93	

Smoking habit	14 (16.9%)	17 (18.3%)	0.96

DBP at week 12	67.5 (7.9)	67.0 (6.1)	0.61
	70.0 (50.0; 85.0)	70.0 (55.0; 85.0)	
	*n* = 83	*n* = 93	

SBP at week 12	114.6 (10.8)	112.7 (10.1)	0.19
	115.0 (90.0; 135.0)	110.0 (90.0; 135.0)	
	*n* = 83	*n* = 93	

Gestational length at inclusion	12.7 (1.5)	12.8 (1.7)	0.94
	12.6 (9.4; 16.9)	12.6 (8.9; 17.7)	
	*n* = 83	*n* = 93	

Extra Mg (mg/day in multivitamin tablets)	31.7 (47.5)	33.9 (48.3)	0.70
	0.0 (0.0; 150.0)	0.0 (0.0; 150.0)	
	*n* = 81	*n* = 92	

Mg intake (n)			

0 (no)	53 (65.4%)	58 (63.0%)	

1 (yes)	28 (34.6%)	34 (37.0%)	0.87

For categorical variables, *n* (%) is presented.

For continuous variables, mean (SD)/median (min; max)/*n* is presented.

For comparison between groups, Fisher's exact test (lowest 1-sided *p* value multiplied by 2) was used for dichotomous variables and the Mann–Whitney *U* test was used for continuous variables.

**Table:** (a) Primary outcome. Increase from gestational week 12 to labour in diastolic (DBP) and systolic (SBP) blood pressure

Variable	Magnesium (*n* = 83)	Placebo (*n* = 93)	*p* value
Increase in DBP ≥15 or SBP ≥30 mm Hg			

0 (no)	46 (56.1%)	56 (60.2%)	

1 (yes)	36 (43.9%)	37 (39.8%)	0.69

Increase in DBP of ≥15 mm Hg (maximum increase)			

0 (no)	47 (57.3%)	57 (61.3%)	

1 (yes)	35 (42.7%)	36 (38.7%)	0.70

Maximum increase in DBP	12.4 (8.3)	10.6 (9.4)	0.17
	10.0 (-5.0; 35.0)	10.0 (-10.0; 30.0)	
	*n* = 83	*n* = 93	

Increase in SBP of ≥30 mm Hg (maximum increase)			

0 (no)	77 (93.9%)	84 (90.3%)	

1 (yes)	5 (6.1%)	9 (9.7%)	0.56

Maximum increase in SBP	12.6 (10.7)	12.9 (11.7)	0.96
	10.0 (-10.0; 55.0)	10.0 (-5.0; 60.0)	
	*n* = 83	*n* = 93	

Gestational HT			

0 (no)	62 (75.6%)	78 (83.9%)	

1 (yes)	20 (24.4%)	15 (16.1%)	0.24

For categorical variables, *n* (%) is presented.

For continuous variables, mean (SD)/median (min; max)/*n* is presented.

For comparison between groups, Fisher's exact test (lowest 1-sided *p* value multiplied by 2) was used for dichotomous variables and the Mann–Whitney *U* test was used for continuous variables.

**Table:** (b) Primary outcome. Increase from gestational week 12 to labour in diastolic blood pressure (DBP) and systolic blood pressure (SBP). Women taking extra Mg were excluded

Variable	Magnesium (*n* = 53)	Placebo (*n* = 58)	*p* value
Increase in DBP ≥15 or SBP ≥30 mm Hg			

**0 **	27 (50.9%)	32 (55.2%)	

**1 **	26 (49.1%)	26 (44.8%)	0.80

Increase in DBP of ≥15 mm Hg (maximum increase)			

**0 **	28 (52.8%)	33 (56.9%)	

**1 **	25 (47.2%)	25 (43.1%)	0.81

Maximum increase in DBP	12.8 (8.0)	11.6 (8.4)	0.34
	10.0 (-5.0; 30.0)	10.0 (0.0; 30.0)	
	*n* = 53	*n* = 58	

Increase in SBP of ≥30 mm Hg (maximum increase)			

**0 **	50 (94.3%)	52 (89.7%)	

**1 **	3 (5.7%)	6 (10.3%)	0.58

Maximum increase in SBP	12.5 (9.0)	12.4 (11.2)	0.70
	10.0 (0.0; 35.0)	10.0 (-5.0; 50.0)	
	*n* = 53	*n* = 58	

Gestational HT			

**0**	42 (79.2%)	48 (82.8%)	

**1 **	11 (20.8%)	10 (17.2%)	0.82

For categorical variables, *n* (%) is presented.

For continuous variables, mean (SD)/median (min; max)/*n* is presented.

For comparison between groups, Fisher's exact test (lowest 1-sided *p* value multiplied by 2) was used for dichotomous variables and the Mann–Whitney *U* test was used for continuous variables.

2018-04-26 analys.sas.

**Table 3:** Labour and fetal outcomes. Missing results are due to labour at other hospitals and no access to medical records (PP).

Variable	Magnesium (*n* = 83)	Placebo (*n* = 93)	*p* value
Gestational length at birth	40.2 (2.0)	39.9 (1.5)	**0.03**
	40.7 (29.3; 42.4)	40.1 (35.0; 42.4)	
	*n* = 80	*n* = 89	

Premature labour (<37+0 weeks)	5 (6.3%)	3 (3.4%)	0.60

Duration of labour (active labour in hours)	7.27 (3.81)	7.07 (3.60)	0.85
	7.00 (1.00; 18.00)	7.00 (1.00; 17.00)	
	*n* = 63	*n* = 74	

Mode of delivery			

Emergency Cesarean Section	14 (17.9%)	7 (8.1%)	0.10

Elective Cesarean Section	1 (1.3%)	5 (5.8%)	0.26

Normal vaginal delivery	59 (75.6%)	62 (72.1%)	0.76

Vacuum extraction	4 (5.1%)	12 (14.0%)	0.10

Blood loss, mL	499.7 (351.5)	518 (347)	0.48
	400.0 (50.0; 2200.0)	425 (150; 2000)	
	*n* = 78	*n* = 86	

Birth weight (g)	3482 (597)	3511 (454)	0.99
	3570 (1335; 4594)	3575 (2540; 4860)	
	*n* = 79	*n* = 86	

Apgar score at 5 minutes	9.73 (0.80)	9.73 (0.64)	0.72
	10.00 (5.00; 10.00)	10.00 (7.00; 10.00)	
	*n* = 78	*n* = 86	

Umbilical arterial pH	7.25 (0.08)	7.25 (0.09)	0.92
	7.27 (7.08; 7.45)	7.26 (7.03; 7.43)	
	*n* = 72	*n* = 80	

NICU care			

0 (no)	62 (80.5%)	76 (88.4%)	

1 (yes)	15 (19.5%)	10 (11.6%)	0.24

For categorical variables, *n* (%) is presented.

For continuous variables, mean (SD)/median (min; max)/*n* is presented.

For comparison between groups, Fisher's exact test (lowest 1-sided *p* value multiplied by 2) was used for dichotomous variables and Chi-square test was used for nonordered categorical variables and the Mann–Whitney *U* test was used for continuous variables.

**Table 4:** Blood levels of Mg, CRP, calcium, albumin, uric acid, cystatin C, IL-6, creatinine, and glomerular filtration rate (GFR) at gestational weeks 12 and 35 and change in levels (Δ) from week 12 to week 35 (PP).

Variable	Magnesium (*n* = 83)	Placebo (*n* = 93)	*p* value
Mg 12	0.78 (0.06)/0.78	0.77 (0.04)/0.78 (0.68; 0.87)	0.30
Reference interval 0,71–0,94 mmol/L	(0.66; 0.92) *n* = 82	*n* = 90	

Mg 35	0.75 (0.06)/0.76	0.73 (0.05)/0.73 (0.62; 0.86)	0.08
	(0.63; 0.90) *n* = 56	*n* = 69	

Δ Mg 12–35	-0.04 (0.05)/-0.05	-0.04 (0.06)/-0.04 (-0.21; 0.06)	0.72
	(-0.16; 0.08) *n* = 56	*n* = 67	

CRP 12	5.45 (4.80)/4.00	4.70 (3.88)/3.00 (2.00; 23.00)	0.26
	(2.00; 26.00) *n* = 82	*n* = 90	

CRP 35	5.98 (5.14)/4.00	5.38 (4.14)/4.00 (2.00; 25.00)	0.74
	(2.00; 28.00) *n* = 55	*n* = 69	

Δ CRP 12–35	0.62 (5.52)/0.00	0.82 (4.50)/0.00 (-21.00; 20.00)	0.27
	(-11.00; 25.00) *n* = 55	*n* = 67	

Calcium 12	2.32 (0.08)/2.31	2.31 (0.08)/2.31 (2.08; 2.58)	0.52
	(2.12; 2.54) *n* = 81	*n* = 90	

Calcium 35	2.29 (0.10)/2.30	2.28 (0.08)/2.27 (2.11; 2.46)	0.32
	(2.08; 2.51) *n* = 55	*n* = 69	

Δ calcium 12–35	-0.02 (0.11)/0.01	-0.03 (0.12)/-0.04 (-0.26; 0.25)	0.44
	(-0.26; 0.24) *n* = 54	*n* = 68	

Albumin 12	37.6 (2.7)/38.0 (30.0;	37.4 (2.5)/37.0 (31.0; 43.0)	0.42
	44.0) *n* = 80	*n* = 89	

Albumin 35	30.8 (2.2)/31.0 (25.0;	30.8 (2.3)/31.0 (26.0; 37.0)	0.73
	36.0) *n* = 55	*n* = 69	

Δ albumin 12-35	-7.00 (2.85)/-7.00	-6.69 (3.03)/-6.00 (-13.00; 2.00)	0.49
	(-13.00; 1.00) *n* = 54	*n* = 67	

Uric acid 12	199.6 (40.2)/200.0	198.4 (39.9)/	0.96
	(103.0; 320.0) *n* = 80	198.0 (99.0; 286.0) *n* = 91	

Uric acid 35	266.7 (66.8)/265.0	276.5 (63.0)/271.0 (153.0;	0.36
	(140.0; 460.0) *n* = 56	490.0) *n* = 67	

Δ Uric acid 12–35	70.7 (54.5)/71.0 (-34.0;	78.5 (49.8)/73.5 (-40.0; 232.0)	0.35
	219.0) *n* = 55	*n* = 66	

Cystatin C 12	0.596 (0.10)/0.59	0.580 (0.10)/0.56 (0.40; 0.89)	0.19
	(0.36; 0.85) *n* = 77	*n* = 81	

Cystatin C 35	0.99 (0.19)/0.97	1.05 (0.31)/1.02 (0.64; 2.61)	0.34
	(0.62; 1.51) *n* = 51	*n* = 59	

Δ C 12–35	0.41 (0.18)/0.40	0.47 (0.29)/0.42 (0.01; 1.74)	0.31
	(0.16; 1.03) *n* = 47	*n* = 51	

IL-6 12	6.72 (8.48)/3.60	7.76 (11.30)/4.65 (0.50; 96.00)	0.33
	(0.70; 50.00) *n* = 79	*n* = 88	

IL-6 35	11.7 (10.0)/8.3 (1.9; 55.0)	12.7 (10.6)/9.7 (2.9; 73.0)	0.32
	*n* = 51	*n* = 67	

Δ IL-6 12–35	5.97 (8.92)/4.05 (-7.00;	4.72 (14.67)/4.50 (-79.00;	0.80
	36.10) *n* = 48	52.00) *n* = 63	

Creatinine 12	50.8 (6.7)/51.0 (39.0; 76.0)	50.3 (8.0)/49.0 (34.0; 82.0)	0.58
	*n* = 82	*n* = 91	

Creatinine 35	50.6 (7.4)/52.0 (35.0; 69.0)	49.9 (9.1)/48.0 (36.0; 95.0)	0.33
	*n* = 56	*n* = 69	

Δ creatinine 12–35	-0.59 (6.77)/-1.000	-1.03 (6.55)/-1.00 (-15.00;	0.55
	(-22.0;24.0) *n* = 56	24.00) *n* = 68	

GFR 12	177.2 (46.7)/171.5	182.1 (43.7)/185.5	0.24
	(101.0; 345.0) *n* = 78	(94.0; 299.0) *n* = 80	

GFR 35	86.3 (21.7)/84.0	82.1 (24.8)/78.0 (20.0; 151.0)	0.26
	(44.0; 138.0) *n* = 52	*n* = 59	

Δ GFR 12–35	-95.6 (45.4)/-84.0	-97.9 (35.1)/-97.5 (-168.0; -	0.29
	(-229.0; -27.0) *n* = 49	15.0) *n* = 50	

For continuous variables, mean (SD)/median (min; max)/*n* is presented.

For comparison between groups, the Mann–Whitney *U* test was used for continuous variables.

## Data Availability

Data (as an Excel file) are available on request from the corresponding author.

## References

[1] Roccella E. J. (2000). Report of the national high blood pressure education program working group on high blood pressure in pregnancy. *American Journal of Obstetrics & Gynecology*.

[2] Antza C., Cifkova R., Kotsis V. (2017). Hypertensive complications of pregnancy: a clinical overview. *Metabolism*.

[3] Roberts J. M., Hubel C. A. (2009). The two stage model of preeclampsia: variations on the theme. *Placenta*.

[4] Young B. C., Levine R. J., Karumanchi S. A. (2010). Pathogenesis of preeclampsia. *Annual Review of Pathology: Mechanisms of Disease*.

[5] Redman C. V., Sargent I. L. (2003). Pre-eclampsia, the placenta and the maternal systemic inflammatory response - a review. *Placenta*.

[6] Anderson U. D., Olsson M. G., Kristensen K. H., Åkerström B., Hansson S. R. (2012). Review: biochemical markers to predict preeclampsia. *Placenta*.

[7] Xiao J. P., Yin Y. X., Gao Y. F. (2012). The increased maternal serum levels of IL-6 are associated with the severity and onset of preeclampsia. *Cytokine*.

[8] Kristensen K., Wide-Swensson D., Schmidt C. (2007). Cystatin C, *β*-2-microglobulin and *β*-trace protein in pre-eclampsia. *Acta Obstetricia et Gynecologica Scandinavica*.

[9] Kristensen K., Larsson I., Hansson S. R. (2007). Increased cystatin C expression in the pre-eclamptic placenta.. *Molecular Human Reproduction*.

[10] Cemgil Arikan D., Aral M., Coskun A., Ozer A. (2012). Plasma IL-4, IL-8, IL-12, interferon-*γ* and CRP levels in pregnant women with preeclampsia, and their relation with severity of disease and fetal birth weight. *The Journal of Maternal-Fetal and Neonatal Medicine*.

[11] Chappell L. C., Seed P. T., Briley A. L. (1999). Effect of antioxidants on the occurrence of pre-eclampsia in women at increased risk: a randomised trial. *The Lancet*.

[12] Bodnar L. M., Tang G., Ness R. B., Harger G., Roberts J. M. (2006). Periconceptional multivitamin use reduces the risk of preeclampsia. *American Journal of Epidemiology*.

[13] Bodnar L. M., Catov J. M., Simhan H. N., Holick M. F., Powers R. W., Roberts J. M. (2007). Maternal vitamin D deficiency increases the risk of preeclampsia. *The Journal of Clinical Endocrinology & Metabolism*.

[14] Purswani J. M., Gala P., Dwarkanath P., Larkin H. M., Kurpad A., Mehta S. (2017). The role of vitamin D in pre-eclampsia: A systematic review. *BMC Pregnancy and Childbirth*.

[15] Wen S. W., Chen X.-K., Rodger M. (2008). Folic acid supplementation in early second trimester and the risk of preeclampsia. *American Journal of Obstetrics & Gynecology*.

[16] Rolnik D. L., Wright D., Poon L. C. (2017). Aspirin versus Placebo in Pregnancies at High Risk for Preterm Preeclampsia. *The New England Journal of Medicine*.

[17] Tolcher M. C., Chu D. M., Hollier L. M. (2017). Impact of USPSTF recommendations for aspirin for prevention of recurrent preeclampsia. *American Journal of Obstetrics & Gynecology*.

[18] Roberge S., Nicolaides K., Demers S., Hyett J., Chaillet N., Bujold E. (2017). The role of aspirin dose on the prevention of preeclampsia and fetal growth restriction: systematic review and meta-analysis. *American Journal of Obstetrics & Gynecology*.

[19] Osikoya O., Jaini P. A., Nguyen A., Valdes M., Goulopoulou S. (2017). Effects of low-dose aspirin on maternal blood pressure and vascular function in an experimental model of gestational hypertension. *Pharmacological Research*.

[20] Zhang W., Iso H., Ohira T., Date C., Tamakoshi A. (2012). Associations of dietary magnesium intake with mortality from cardiovascular disease: The JACC study. *Atherosclerosis*.

[21] Porr PJ., Nechifor M., Durlach J. (2006). Advances in magnesium research. *John Libbey Eurotext*.

[22] Kass L., Weekes J., Carpenter L. (2012). Effect of magnesium supplementation on blood pressure: A meta-analysis. *European Journal of Clinical Nutrition*.

[23] Makrides M., Crosby D. D., Bain E., Crowther C. A. (2014). Magnesium supplementation in pregnancy. *Cochrane Database of Systematic Reviews*.

[24] Enaruna N. O., Ande A., Okpere E. E. (2013). Clinical significance of low serum magnesium in pregnant women attending the University of Benin Teaching Hospital. *Nigerian Journal of Clinical Practice*.

[25] Nielsen T. F., Rylander R. (2011). Urinary calcium and magnesium excretion relates to increase in blood pressure during pregnancy. *Archives of Gynecology and Obstetrics*.

[26] Bullarbo M., Ödman N., Nestler A. (2013). Magnesium supplementation to prevent high blood pressure in pregnancy: A randomised placebo control trial. *Archives of Gynecology and Obstetrics*.

[27] Nestler A., Rylander R., Kolisek M. (2014). Blood pressure in pregnancy and magnesium sensitive genes. *Pregnancy Hypertension: An International Journal of Women's Cardiovascular Health*.

[28] Rylander R. (2014). Magnesium in pregnancy blood pressure and pre-eclampsia - A review. *Pregnancy Hypertension: An International Journal of Women's Cardiovascular Health*.

[29] Bullarbo M., Rylander R. (2015). Diastolic blood pressure increase is a risk indicator for pre-eclampsia. *Archives of Gynecology and Obstetrics*.

[30] Khedun S. M., Ngotho D., Moodley J., Naicker T. (1998). Plasma and red cell magnesium levels in black African women with hypertensive disorders of pregnancy. *Hypertension in Pregnancy*.

[31] Schoenaker D., Soedamah-Muthu S. S., Mishra G. D. (2014). The association between dietary factors and gestational hypertension and pre-eclampsia: A systematic review and meta-analysis of observational studies. *BMC Medicine*.

[32] Spätling L., Classen H. G., Kisters K. (2017). Supplementation of magnesium in pregnancy. *Journal of Pregnancy and Child Health*.

[33] Ismail Y., Ismail A. A., Ismail A. A. A. (2010). The underestimated problem of using serum magnesium measurements to exclude magnesium deficiency in adults; A health warning is needed for “normal” results. *Clinical Chemistry and Laboratory Medicine*.

[34] Sibai B. M., Villar M. A., Bray E. (1989). Magnesium supplementation during pregnancy: A double-blind randomized controlled clinical trial. *American Journal of Obstetrics & Gynecology*.

[35] Rylander R. (2014). Bioavailability of magnesium salts - A review. *Journal of Pharmacy and Nutrition Sciences*.

